# Evaluation des protocoles analgésiques pour la prise en charge de la douleur au cours de la lithotripsie extra corporelle

**DOI:** 10.11604/pamj.2019.32.109.17690

**Published:** 2019-03-08

**Authors:** Kheireddine Mrad Daly, Kays Chaker, Sami Ben Rhouma, Mohamed Ali Ben Chehida, Yassine Ouanes, Ahmed Sellami, Yassine Nouira

**Affiliations:** 1 Service d’Urologie, Hôpital La Rabta, Faculté de Médecine de Tunis, Université de Tunis, El Manar, Tunisie

**Keywords:** Lithiases urinaires, lithotritie extracorporelle, échelle visuelle analogique, Urinary lithiasis, extracorporeal shock wave lithotripsy, visual analog scale

## Abstract

La douleur ressentie au cours d'une séance de lithotritie extracorporelle (LEC) est le facteur limitant le plus important de cette technique. Le but de notre travail était de comparer l'efficacité des différents types d'analgésiques utilisés pour le contrôle de la douleur pendant les séances de LEC. Nous avons mené une étude prospective colligeant 300 patients présentant une lithiase urinaire justifiant un traitement par LEC. Les patients ont été répartis de façon randomisée en trois groupes: le groupe I, incluant 100 patients ayant reçu 2cc de sérum physiologique en IM (intra musculaire) (placebo), le groupe II regroupant 100 patients ayant reçu 100mg de kétoprofène en IM tandis que le groupe III colligeant 100 patients ayant eu une application locale d'une crème contenant la lidocaïne et la prilocaïne. L'échelle visuelle analogique (EVA) a servi à évaluer la douleur à 10 minutes et à la fin de la séance. L'EVA moyenne à 10 minutes et à la fin de la LEC étaient respectivement de 3,7 et 4,91. Aucune différence significative n'a été trouvée entre les trois groupes concernant: les données épidémiologiques (âge, sexe, IMC, antécédents pathologies) et les caractéristiques du calcul (côté, taille, localisation, présence ou non de sonde double J). L'interruption précoce de la séance de LEC a été notée chez 11 patients du groupe I, avec une différence significative par rapport aux autres groupes (p=0,003). L'EVA à 10 minutes et à la fin de séance de LEC, était statistiquement plus élevée dans le groupe I par rapport aux groupes II et III (p < 0,001). Aussi, la LEC était nettement plus efficace dans les groupes (II et III) comparativement au groupe I (p<0,001). L'utilisation d'un traitement antalgique est nécessaire lors de la séance de lithotritie extracorporelle. Les deux molécules antalgiques évaluées ont montré un bon contrôle de la douleur ainsi qu'une augmentation de l'efficacité du traitement par lithotritie.

## Abstract (en)

Extracorporeal shock wave lithotripsy-related pain is the largest limiting factor in this technique. Our study aimed to compare the effectiveness of different types of analgesics for pain management used during ESWL sessions. We conducted a prospective study of 300 patients with urinary lithiasis justifying ESWL treatment. The patients were randomized to three groups: group I, included 100 patients who received intramuscular injection of 2cc of physiological saline solution (placebo), group II included 100 patients who received intramuscular injection of ketoprofen 100mg while group III included 100 patients who received lidocaine and prilocaine topical cream. Visual Analog Scale (VAS) was used to assess pain 10 minutes after and at the end of the session. Mean VAS score 10 minutes after and at the end of ESWL session was 3.7 and 4.91 respectively. There was no significant difference among the three groups with respect to: epidemiological data (age, sex, BMI, patient’s history) and the characteristics of the renal stone (side, size, location, presence or not of double-J ureteral catheter). Eleven patients in the Group I terminated treatment early, with a significant difference compared to the other groups (p=0.003). VAS score 10 minutes after and at the end of ESWL session was statistically higher in Group I compared to Groups II and III (p < 0.001). Moreover, ESWL session was significantly more effective in Groups (II and III) compared to Group I (p<0.001). Pain treatment is necessary during ESWL sessions. Two painkillers molecules were assessed, which showed good pain control as well as an increase in the effectiveness of lithotripsy.

## Introduction

La lithotritie extracorporelle (LEC) est une approche non chirurgicale dans la thérapeutique de la lithiase urinaire. Elle représente actuellement le traitement de première intention de plus de 50% des calculs rénaux et urétéraux [1]. L'évolution des appareils de lithotritie tend à améliorer la qualité du repérage des calculs, le contrôle de la fragmentation et le confort du patient, tout en réduisant le recours à l'anesthésie. La collaboration des patients est un critère majeur pour l'efficacité du traitement, ce qui rend le choix de l'anesthésie très important. Actuellement, il n'existe pas de protocole standardisé sur le type d'analgésie à suivre, permettant à la fois un meilleur contrôle de la douleur avec moins d'effets indésirables. Nous présentons les résultats d'une étude prospective, randomisée, comparant l'analgésie par kétoprofène en intramusculaire à l'analgésie par application cutanée de lidocaïne associée à la prilocaïne, contre placebo.

## Méthodes

Il s'agit d'une étude prospective, randomisée, contrôlée, incluant 300 patients traités par LEC, entre juin 2016 et mars 2017. Nous avons inclus les patients âgés de 18 ans et plus, présentant un calcul rénal ou urétéral lombaire justifiant une LEC selon les recommandations du Comité Lithiase de l'Association Française d'Urologie [2]. Un consentement écrit avant d'initier l'étude a été signé par chaque participant. Les lithiases étaient toutes radio-opaques et de grand axe compris entre 5 et 25 mm. Nous n'avons pas inclus dans cette étude les patients présentant: une contre-indication à la LEC, une maladie chronique nécessitant un traitement antalgique au long cours ou une lithiase radio-transparente de taille supérieure à 2,5 cm et de localisations autres que rénale ou urétérale lombaire. N'ont pas été inclus également les patients qui ont déjà participé à cette même étude et ceux présentant une intolérance ou une contre-indication à l'une des molécules utilisées. Nous avons exclu, par la suite, les patients présentant des troubles auditifs, visuels ou de compréhension ne leurs permettant pas de répondre aux questions et les patients considérés comme perdus de vue n'ayant pas consulté après la séance de LEC. Les patients ont eu initialement un examen physique, un dosage de la créatinine sérique et un bilan d'hémostase, un examen cytobactériologique des urines, un cliché de l'arbre urinaire sans préparation (AUSP) et une échographie de l'appareil urinaire, une urographie intraveineuse ou un scanner abdominopelvien. La taille de la lithiase a été calculée selon son plus grand diamètre sur le cliché d'AUSP. Durant la période de l'étude, 383 patients ont été colligés. Au final 300 patients ont été retenus. La cadence de tir ainsi que la durée de la séance de lithotritie ont été fixées de façon identique pour tous les patients. De même, la progression de la séance a été effectuée par palier de puissance croissante, selon une séquence prédéfinie, pour tous les malades.

Les patients ont été randomisés de façon aléatoire en 3 groupes: **groupe I**: patients ayant reçu 2cc de sérum physiologique en IM (groupe témoin/placebo); **groupe II**: patients ayant reçu 100mg de Kétoprofène en IM; **groupe III**: patients ayant eu une application cutanée locale, sur la peau du flanc du côté de la lithiase, d'une crème contenant un mélange équimolaire de lidocaïne (25mg/g) et de prilocaïne (25mg/g). L'application était faite en couche épaisse, une heure avant la LEC. Le site d'application de la crème a été recouvert par un pansement occlusif. L'analgésie a été administrée 30 min avant le début de la séance pour les groupes I et II, et une heure avant pour le groupe III. Cette étude a été menée en simple aveugle, le patient n'avait pas connaissance de la molécule reçue. Le critère de jugement principal était l'évaluation de la douleur par l'Échelle Visuelle Analogique (EVA). La mesure était effectuée à 10 minutes du début de la séance de LEC (temps T1), et à la fin de la séance de 35 minutes (temps T2). Si la douleur n'était plus supportable par le patient, la séance était arrêtée, et l'EVA était chiffrée à 10. Le critère de jugement secondaire était l'évaluation de l'efficacité de la LEC, sur un AUSP de contrôle réalisé au minimum deux semaines après la séance. Les critères d'interprétation sont résumés sur le Tableau 1. Dans tous les tests statistiques, le seuil de signification a été fixé à 0,05.

**Tableau 1:** interprétation du résultat de la LEC

Possibilités	Interprétation	Résultat
Sans fragment résiduel	Le calcul est fragmenté et éliminé en totalité.	Succès de la LEC: bon résultat
Fragmentation totale et élimination partielle	Le calcul est fragmenté totalement (fragments<4mm) mais persistent des fragments dont la somme est > 4mm.	Succès de la LEC: bon résultat
Fragmentation partielle et élimination partielle	Le calcul est fragmenté partiellement (fragments>4mm) avec persistance de fragments dont la somme est >4mm.	Résultat intermédiaire
Fragmentation partielle sans élimination	Le calcul est fragmenté partiellement sans être éliminé.	Mauvais résultat
Le calcul n’est pas du tout fragmenté ni éliminé	Même aspect du calcul après la séance de LEC	Mauvais résultat

## Résultats

La population étudiée était composée de 184 hommes et de 116 femmes (Sex-Ratio=1,58). L'âge moyen était de 53,67 ans (23 à 80 ans). L'indice de masse corporelle (IMC) moyen était de 25,34 kg/m^2^ avec des extrêmes allant de 19 à 35 kg/m^2^. La localisation des lithiases du côté gauche était légèrement prédominante (55%). La taille moyenne des calculs était de 11,6 mm (5-22 mm). Les calculs étaient essentiellement de siège rénal (pyélique ou caliciel) dans 87,3% des cas. Pour les patients des trois groupes confondus, l'EVA douleur moyenne était de 3,7 à 10 minutes du début de la séance de LEC (temps T1), et de 4,91 à la fin de celle-ci (temps T2). Nous n'avons pas trouvé de différence statistiquement significative entre les 3 groupes de patients selon l'âge, le sexe, l'IMC, les antécédents pathologiques et les antécédents de LEC antérieures (Tableau 2). Aussi, nous n'avons pas trouvé de différence statistiquement significative entre les 3 groupes de patients selon le côté, la taille et la localisation des calculs ainsi que la présence ou non de sonde double J lors de la séance de LEC (Tableau 3). On n'avait pas noté des complications graves lors du déroulement de la séance de LEC. L'interruption précoce de la séance de LEC, causée par une douleur devenue intolérable, a été notée chez 11 patients du groupe I (placebo) et seulement chez 2 patients dans chacun des groupes II et III (p=0,003). L'EVA à 10 min du début de la LEC était statistiquement plus élevée dans le groupe placebo que dans le groupe kétoprofène et le groupe lidocaïne + prilocaïne crème (respectivement p<0,001 et p<0,001) (Tableau 4). La comparaison entre les groupes II et III a montré une diminution de la douleur au profit du groupe II, sans différence statistiquement significative (p = 0,085). L'EVA à la fin de la séance était statistiquement plus élevée dans le groupe placebo que dans les groupes II et III (respectivement p<0,001 et p<0,001). Les patients du groupe II ont eu moins mal, à la fin de la séance, que ceux du groupe III, mais ce résultat n'était pas statistiquement significatif (p = 0,325) (Tableau 4). Concernant l'efficacité du traitement par LEC, nous avons trouvé un « bon résultat » (fragmentation totale du calcul avec élimination complète ou partielle) de façon statistiquement plus importante dans les groupes sous traitements (II et III) par rapport au groupe placebo (I) (p=0,04). Cependant, nous n'avons pas noté de différence significative entre le groupe II (kétoprofène) et le groupe III (lidocaïne+prilocaïne crème) selon ce paramètre (bon résultat), bien qu'il soit plus élevé dans groupe II (p=0,2) (Tableau 5).

**Tableau 2:** les différentes caractéristiques des patients selon les trois groupes

Paramètres	Groupe 1 (N = 100)	Groupe 2 (N = 100)	Groupe 3 (N = 100)	Valeur de p
**Sexe**				
Masculin	61	60	63	0,906
Féminin	39	40	37	
Age	54,23	52,04	54,73	0,283
**Index de la masse corporelle (IMC)**				
Normal	51	52	60	0,698
Surpoids	33	32	25	
Obésité	16	16	15	
**Antécédents pathologiques**				
Oui	56	54	49	0,593
Non	44	46	51	
**Hypertension artérielle**				
Oui	24	19	26	0,480
Non	76	81	74	
**Diabète**				
Oui	23	23	27	0,982
Non	77	77	76	
**Lombotomie**				
Oui	12	11	10	0,903
Non	82	89	90	
**Antécédents de LEC**				
Oui	65	68	62	0,673
Non	35	32	38	

**Tableau 3:** répartition des patients selon les caractéristiques de la lithiase

Paramètres	Groupe 1 (N= 100)	Groupe 2 (N = 100)	Groupe 3 (N = 100)	Valeur de p
**Côté**				
Droit	48	48	39	0,336
Gauche	42	52	61	
**Taille**	11,69	11,09	12,04	0,256
**Localisation**				
Pyélon	33	23	33	0,362
Calice supérieur	17	18	8	
Calice Moyen	13	19	15	
Calice inférieur	27	27	29	
Uretère	10	13	15	
**Présence de sonde JJ**				
Oui	18	16	18	0,911
Non	82	84	82	

**Tableau 4:** EVA des patients

Paramètres	Groupe I (N=100)	Groupe II (N = 100)	Groupe III (N = 100)	Valeur de p
EVA à 10 minutes	5,07 ± 1,68	2,85 ± 1,88	3,18 ± 1,66	**<0,001**
EVA fin de la LEC	6,4 ± 1,80	4,02 ± 2,065	4,3 ± 1,806	**<0,001**

**Tableau 5:** résultat de la LEC pour les trois groupes

Paramètres	Groupe I (N=100)	Groupe II (N=100)	Groupe III (N=100)	Valeur de p
Sans Fragment	17	42	30	
Fragmentation totale + élimination partielle	34	27	26	
**Bon Résultat**	**51**	**69**	**56**	**0,004**
Fragmentation partielle + élimination partielle	23	24	33	
**Résultat intermédiaire**	23	24	33	
Fragmentation partielle + pas d’élimination	9	5	7	
Pas de fragmentation ni d’élimination	17	2	4	
**Mauvais résultat**	**26**	**7**	**11**	
**Tableau 5:** résultat de la LEC pour les trois groupes				
**Paramètres**	**Groupe I (N=100)**	**Groupe II (N=100)**	**Groupe III (N=100)**	**Valeur de p**
Sans Fragment	17	42	30	
Fragmentation totale + élimination partielle	34	27	26	
**Bon Résultat**	**51**	**69**	**56**	**0,004**
Fragmentation partielle + élimination partielle	23	24	33	
**Résultat intermédiaire**	23	24	33	
Fragmentation partielle + pas d’élimination	9	5	7	
Pas de fragmentation ni d’élimination	17	2	4	
**Mauvais résultat**	**26**	**7**	**11**	
**Tableau 5:** résultat de la LEC pour les trois groupes				
**Paramètres**	**Groupe I (N=100)**	**Groupe II (N=100)**	**Groupe III (N=100)**	**Valeur de p**
Sans Fragment	17	42	30	
Fragmentation totale + élimination partielle	34	27	26	
**Bon Résultat**	**51**	**69**	**56**	**0,004**
Fragmentation partielle + élimination partielle	23	24	33	
**Résultat intermédiaire**	23	24	33	
Fragmentation partielle + pas d’élimination	9	5	7	
Pas de fragmentation ni d’élimination	17	2	4	
**Mauvais résultat**	**26**	**7**	**11**	

## Discussion

Le premier lithotripteur pour le traitement des calculs urinaires, le HM1 (Human Model 1, Dornier, Allemagne), a été introduit en 1980 par Chaussy *et al.* [3]. Malgré les nouvelles avancées thérapeutiques, telles que la néphrolithotomie percutanée et les techniques endoscopiques, plus que 50% des lithiases sont encore traitées par LEC [1]. La LEC a connu des modifications importantes depuis sa première description et l'approche de l'anesthésie a considérablement changé depuis. L'anesthésie générale et régionale a laissé place aux techniques analgésiques sédatives permettant la réalisation de la lithotritie en ambulatoire actuellement [4]. Mais la douleur reste la complication et le facteur limitant le plus important de la lithotritie. Une mauvaise analgésie lors de la LEC peut diminuer la focalisation des ondes de chocs sur le calcul et ainsi diminuer l'efficacité de la lithotritie. Aussi, les patients sans douleur sont les meilleurs candidats pour la LEC en augmentant l'efficacité de la procédure [5]. Différents agents analgésiques, y compris les opioïdes, les AINS, l'analgésie locale et différentes combinaisons, sont utilisés avec différents modes d'administration. Cependant, il n'y a pas encore de consensus sur l'analgésie standard pour la douleur lors LEC [6,7]. Les opioïdes sont les antalgiques les plus couramment étudiés au cours de la LEC. Diverses techniques d'administration ont été utilisées, telles que le bolus IM ou IV et plus récemment l'analgésie contrôlée par le patient. Ils ont un effet antalgique très efficace, mais au prix d'effets secondaires multiples (dépression du système nerveux central, insuffisance circulatoire ou respiratoire, nausée vomissements…), nécessitant un monitorage continue et l'implication de médecins anesthésistes réanimateurs. Ceci limite leur utilisation en ambulatoire [8]. Initialement, les AINS et les crèmes anesthésiques topiques ont été utilisés en association avec des opioïdes pour réduire leurs doses et leurs effets indésirables. Cependant, dans certains centres actuellement, ils ont remplacé les opioïdes et ont commencé à être utilisés comme agents exclusifs pour soulager la douleur au cours de la LEC. Grace à leur effet analgésique efficace, les AINS sont largement utilisés lors de la LEC [9,10]. En inhibant l'enzyme de la cyclo-oxygénase (COX), ils réduisent la synthèse de prostaglandine qui est un médiateur de l'inflammation et de la douleur. Les AINS doivent être administrés 30 à 60 minutes avant la séance [11]. Leurs effets secondaires comprennent des troubles gastro-intestinaux, des troubles de la coagulation et des réactions d'hypersensibilité [11].

Dans une étude visant à évaluer la douleur lors de la LEC, il a été démontré que les AINS, en monothérapie, étaient suffisant chez 70% des patients [12]. Différents AINS tels que le Piroxicam, Kétorolac, Ténoxicame, et le Diclofénac ont été utilisés dans l'analgésie lors de la LEC en monothérapie ou combinés à d'autres analgésiques. Le Kétoprofène est un anti-inflammatoire non stéroïdien dérivé de l'acide aryl carboxylique, du groupe des propioniques. Il possède les propriétés suivantes: antalgique périphérique et central, antipyrétique, anti-inflammatoire et d'inhibition de courte durée des fonctions plaquettaires. Dans une méta-analyse, parue en 2013 comparant l'efficacité antalgique du kétoprofène à celle de l'ibuprofène et du diclofénac et incluant 898 patients, il a été démontré que l'effet antalgique du kétoprofène est plus efficace que ces deux autres AINS [13]. Plusieurs études ont montré l'efficacité du Kétoprofène dans la diminution de la douleur consécutive à une chirurgie orthopédique ou à des coliques néphrétiques [13]. Mais peu d'études ont évalué l'efficacité antalgique du Kétoprofène lors de la LEC. Tokgoz *et al*. dans une étude publiée en 2010 ont comparé le Kétoprofène au Diclofénac. Les deux molécules ont été injectées par voie IM, 30 minutes avant la séance de LEC. L'EVA de la douleur a été significativement moins importante chez les patients recevant le Kétoprofène [14]. La dose de 50 mg de kétoprofène a été utilisée, et l'EVA moyenne de la douleur chez les patients recevant cette dose était de 4,2. Yesli *et al*. dans une étude randomisée contrôlée publiée en 2014 comparant l'efficacité analgésique de 3 types de médicaments lors de la LEC, ont montré une baisse significative de la douleur chez les patients recevant 50 mg de Kétoprofène en IM [7]. Dans notre étude on a utilisé la dose de 100 mg en IM de Kétoprofène, 30 minutes avant le début de la séance de LEC. L'EVA douleur moyenne, chez les patients recevant cette dose, était de 4,02 à la fin de la séance. Les crèmes analgésiques appliquées localement ont un rôle controversé dans le contrôle de la douleur. Leurs avantages comprennent leur simplicité d'utilisation, leur caractère non invasif, l'évitement des effets secondaires des analgésiques intramusculaires et intraveineux [15].

Plusieurs études ont utilisé un mélange eutectique de crème pour analgésie locale (EMLA) contenant 2,5% de Prilocaïne et 2,5% de Lidocaïne, comme moyen antalgique lors de la LEC, et ceci depuis 1991 [16]. Sa profondeur de pénétration dans la peau intacte est d'environ 4 mm après 60 minutes [17]. Elle doit être appliquée sous un pansement occlusif, 60 à 90 minutes avant la procédure. Le respect du timing est important [17]. Le principal reproche à cette crème est qu'elle a un pouvoir analgésique relativement faible et qu'elle nécessite une analgésie supplémentaire [18]. De ce fait, très peu d'études ont évalué son pouvoir antalgique seul au cours de la LEC. D'autre part, on trouve une grande discordance concernant l'efficacité analgésique de cette crème lors de la LEC dans les différentes publications. Acar *et al*. dans une étude portant sur 60 patients, ont comparé un groupe recevant la crème EMLA associé au Rémifentanil, administré à la demande, à un groupe recevant le Remifentanil avec une crème placebo. Ils n'ont trouvé aucune différence entre les doses de Remifentanil ajoutées et le score de la douleur entre les deux groupes [19]. Eryilderrim *et al*. aussi, dans leur analyse portée sur 120 patients parue en 2009, ont comparé l'effet analgésique lors de la LEC de la crème EMLA^®^ seule, du Diclofenac seul et de leur association. Ils ont montré aucune différence, dans le contrôle de la douleur, chez les patients recevant du Diclofenac avec ou sans crème, et l'effet supérieur du Diclofenac sur EMLA [20]. Néanmoins, ces études sont relativement anciennes, utilisant des lithotripteurs de première ou seconde générations et comparant un nombre réduit de patients. D'autre part, Saita *et al*. ont décrit le rôle de l'anesthésie cutanée associée à un antalgique IM dans la diminution de la douleur et dans l'augmentation du taux de succès de la LEC [21]. Vilar *et al*. dans leur analyse parue en 2012, ont utilisé la Pethidine IV et la crème EMLA dans un groupe et la Pethidine associée à une crème Placebo dans un autre. Ils ont conclu que la crème EMLA, associée à la péthidine, améliorait significativement l'EVA de la douleur ainsi que la fragmentation du calcul comparée au deuxième groupe [22]. De même, Kumar *et al.* ont montré un meilleur contrôle de la douleur, ainsi qu'un taux de stone free supérieur avec la crème EMLA en association avec Diclofenac, par rapport à l'un ou l'autre de ses antalgiques utilisés séparément [10]. Dans notre étude, comparativement au groupe placebo, le groupe ayant reçu cette crème avait une EVA douleur statistiquement plus faible (4,3 vs 6,4; p<0,001) et une efficacité plus importante de la LEC.

## Conclusion

Notre étude a montré que les deux types de médicaments évalués (Kétoprofène et Prilocaïne+Lidocaïne crème) améliorent le confort du patient lors de la LEC, augmentant ainsi l'efficacité de ce traitement des lithiases rénales et urétérales. L'injection intramusculaire de 100 mg de kétoprofène, 30 minutes avant la séance de LEC, a montré une plus grande efficacité analgésique comparativement au placebo et à la crème EMLA. Cette classe thérapeutique (AINS = kétoprofène) étant plus disponible, moins coûteuse et plus facile à utiliser que la crème contenant de la procaïne + lidocaïne, apparait comme l'agent idéal du contrôle de la douleur lors de la LEC dans notre étude. L'association de ces deux antalgiques devrait améliorer encore plus le confort du patient et l'efficacité de la lithotritie. Au vu de nos résultats et des données de la littérature, nous proposons une analgésie systématique avant toute séance de LEC. Surtout chez les sujets minces, présentant des calculs caliciels supérieurs ou une sonde double J. Cette analgésie devrait être élargie aux autres actes urologiques jugés gênants et douloureux par le patient, tels que la cystoscopie et la biopsie prostatique.

### Etat des connaissances actuelles sur le sujet

- La lithiase urinaire est une pathologie fréquente dans le monde et en Tunisie. Elle touche environ 10% de la population générale. Par sa fréquence, ses complications et les dépenses qui en découlent, elle pose un vrai problème de santé publique;
- La douleur est la complication et le facteur limitant le plus important de cette technique. Celle-ci est variable selon les individus et justifie le recours au traitement antalgique;
- Ce traitement diffère largement d'une équipe à une autre, et jusqu'à ce jour, on ne dispose pas de recommandations claires permettant de choisir le traitement antalgique le plus efficace et approprié lors d'une séance de LEC.

### Contribution de notre étude à la connaissance

- Notre étude a montré que les deux types de médicaments évalués (kétoprofène et prilocaïne+lidocaïne crème) améliorent le confort du patient lors de la LEC, augmentant ainsi l'efficacité de ce traitement des lithiases rénales et urétérales. L'injection intramusculaire de 100 mg de kétoprofène, 30 minutes avant la séance de LEC, a montré une plus grande efficacité analgésique comparativement au placebo et à la crème;
- Cette classe thérapeutique (AINS = kétoprofène) étant plus disponible, moins coûteuse et plus facile à utiliser que la crème contenant de la procaïne + lidocaïne, apparait comme l'agent idéal du contrôle de la douleur lors de la LEC dans notre étude;
- Au vu de nos résultats et des données de la littérature, nous proposons une analgésie systématique avant toute séance de LEC. Surtout chez les sujets minces, présentant des calculs caliciels supérieurs ou une sonde double J. Cette analgésie devrait être élargie aux autres actes urologiques jugés gênants et douloureux par le patient, tels que la cystoscopie et la biopsie prostatique.

## Conflits des intérêts

Les auteurs ne déclarent aucun conflit d'intérêts.

## References

[1] Christian C, Thorsten B (2009). The preferred treatment for upper tract stones is extracorporeal shock wave lithotripsy (ESWL) or ureteroscopic: pro ESWL. Urology.

[2] Saussine C, Lechevallier E, Traxer O (2008). Les recommandations ou guidelines de la lithiase urinaire. Prog Urol.

[3] Chaussy C, Schmiedt E, Jocham D, Brendel W, Forssmann B, Walther V (1982). First clinical experience with extracorporeally induced destruction of kidney stones by shock waves. J Urol.

[4] Fredman B, Jedeikin R, Olsfanger D, Aronheim M (1993). The opioid-sparing effect of diclofenac sodium in outpatient extracorporeal shock wave lithotripsy (ESWL). J Clin Anesth.

[5] Young A, Ismail M, Papatsoris A (2012). Entonox^®^ inhalation to reduce pain in common diagnostic and therapeutic outpatient urological procedures: a review of the evidence. Ann R Coll Surg Engl.

[6] Gupta NP, Kumar A (2008). Analgesia for pain control during extracorporeal shock wave lithotripsy: current status. Indian J Urol.

[7] Yesil S, Polat F, Ozturk U (2014). Effect of different analgesics on pain relief during extracorporeal shock wave lithotripsy. Hippokratia.

[8] Demir A, Cecen K, Karadag MA (2015). Pain control using pethidine in combination with diazepam compared to diclofenac in combination with hyoscine-n-butyl bromide: in patients undergoing extracorporeal shock wave lithotripsy. Cent European J Urol.

[9] Dawson C, Vale JA, Corry DA, Cohen N, Gallagher J, Nockler IB (1994). Choosing the correct pain relief for extracorporeal lithotripsy. Br J Urol.

[10] Kumar A, Gupta NP, Hemal AK, Wadhwa P (2007). Comparison of three analgesic regimens for pain control during shockwave lithotripsy using dornier delta compact lithotripter: a randomized clinical trial. J Endourol.

[11] Mezentsev VA (2009). Meta-analysis of the efficacy of non-steroidal anti-inflammatory drugs vs opioids for SWL using modern electromagnetic lithotripters. Int braz j urol.

[12] Gupta NP, Kumar A (2008). Analgesia for pain control during extracorporeal shock wave lithotripsy: current status. Indian J Urol.

[13] Sarzi-Puttini P, Atzeni F, Lanata L, Bagnasco M (2013). Efficacy of ketoprofen vs ibuprofen and diclofenac: a systematic review of the literature and meta-analysis. Clin Exp Rheumatol.

[14] Tokgoz H, Yurtlu S, Hanci V, Turksoy O, Erol B, Akduman B (2010). Comparison of the analgesic effects of dexketoprofen and diclofenac during shockwave lithotripsy: a randomized, double-blind clinical trial. J Endourol.

[15] Honnens de Lichtenberg M, Miskowiak J, Mogensen P (1992). Local anesthesia for extracorporeal shock wave lithotripsy: a study comparing eutetic mixture of local anesthetics cream and lidocaine infiltration. J Urol.

[16] Bierkens AF, Maes RM, Hendrikx JM, Erdos AF, de Vries JD, Debruyne FM (1991). The use of local anesthesia in second generation extracorporeal shock wave lithotripsy: eutectic mixture of local anesthetics. J Urol.

[17] Arendt-Neilsen L, Bjerring P, Nielsen J (1990). Regional variations in analgesic efficacy of EMLA cream: quantitatively evaluated by argon laser stimulation. Acta Derm Venereol.

[18] Basar H, Yilmaz E, Ozcan S, Buyukkocak U, Sari F, Apan A (2003). Four analgesic techniques for shockwave lithotripsy: eutectic mixture local anesthetic is a good alternative. J Endourol.

[19] Acar A, Erhan E, Nuri Deniz M (2013). The effect of EMLA cream on patient-controlled analgesia with remifentanil in ESWL procedure: a placebo-controlled randomized study. Anesth Pain Med.

[20] Eryildirim B, Kuyumcuoglu U, Tarhan F (2009). Comparison of three analgesic treatment protocols for pain management during extracorporeal shock wave lithotripsy. Urol Int.

[21] Saita A, Bonaccorsi A, Aquilino M (2004). ESWL: comparing two analgesic techniques: our experience. Urol Int.

[22] Vilar DG, Fadrique GG, Sacoto CD (2012). Topical EMLA for pain control during extracorporeal shock wave lithotripsy: prospective, comparative, randomized, double-blind study. Urol Res.

